# The Brown Midrib Leaf (*bml*) Mutation in Rice (*Oryza sativa* L.) Causes Premature Leaf Senescence and the Induction of Defense Responses

**DOI:** 10.3390/genes9040203

**Published:** 2018-04-09

**Authors:** Delara Akhter, Ran Qin, Ujjal Kumar Nath, Md. Alamin, Xiaoli Jin, Chunhai Shi

**Affiliations:** 1 Department of Agronomy, Zhejiang University, Hangzhou 310027, China; 11516090@zju.edu.cn (D.A.); ranqin89@zju.edu.cn (R.Q.); alamin@zju.edu.cn (M.A.); jinxl@zju.edu.cn (X.J.); 2 Department of Genetics and Plant Breeding, Sylhet Agricultural University, Sylhet 3100, Bangladesh; 3 Department of Genetics and Plant Breeding, Bangladesh Agricultural University, Mymensingh 2202, Bangladesh; ujjalnath@gmail.com

**Keywords:** rice, *Oryza sativa* L., brown midrib leaf (*bml*), leaf senescence, chlorophyll biosynthesis, gene mapping, defense responses, hormone signaling

## Abstract

Isolating and characterizing mutants with altered senescence phenotypes is one of the ways to understand the molecular basis of leaf aging. Using ethyl methane sulfonate mutagenesis, a new rice (*Oryza sativa*) mutant, brown midrib leaf (*bml*), was isolated from the *indica* cultivar ‘Zhenong34’. The *bml* mutants had brown midribs in their leaves and initiated senescence prematurely, at the onset of heading. The mutants had abnormal cells with degraded chloroplasts and contained less chlorophyll compared to the wild type (WT). The *bml* mutant showed excessive accumulation of reactive oxygen species (ROS), increased activities of superoxide dismutase, catalase, and malondialdehyde, upregulation of senescence-induced STAY-GREEN genes and senescence-related transcription factors, and down regulation of photosynthesis-related genes. The levels of abscisic acid (ABA) and jasmonic acid (JA) were increased in *bml* with the upregulation of some ABA and JA biosynthetic genes. In pathogen response, *bml* demonstrated higher resistance against *Xanthomonas oryzae* pv. *oryzae* and upregulation of four pathogenesis-related genes compared to the WT. A genetic study confirmed that the *bml* trait was caused by a single recessive nuclear gene (*BML*). A map-based cloning using insertion/deletion markers confirmed that *BML* was located in the 57.32kb interval between the *L5IS7* and *L5IS11* markers on the short arm of chromosome 5. A sequence analysis of the candidate region identified a 1 bp substitution (G to A) in the 5′-UTR (+98) of *bml*. *BML* is a candidate gene associated with leaf senescence, ROS regulation, and disease response, also involved in hormone signaling in rice. Therefore, this gene might be useful in marker-assisted backcrossing/gene editing to improve rice cultivars.

## 1. Introduction

Leaf senescence is governed by a finely tuned and multifaceted regulatory network [1]. It brings major changes in the cellular metabolism and gene expression patterns of leaves [2]. These changes allow the controlled breakdown of chlorophyll and the recycling of cellular components. The most striking aspect of such changes in the leaf is yellowing caused by the disintegration of chlorophyll by the action of chlorophyllase (CLH), which converts chlorophyll into chlorophyllide [3]. The visible yellowing reflects chloroplast degradation in mesophyll cells [1]. Later on, CLH takes part in the dissociation of macromolecules such as lipids and nucleic acids, which finally leads to cell death through degradation of mitochondria and nuclei [1]. Premature leaf aging can decrease crop yield [4], because senescent leaves with degraded chlorophyll cannot photosynthesize. Therefore, the maintenance of a proper timing of leaf senescence might enhance a plant's photosynthetic capability and increase crop yield [5]. It is possible for a plant to initiate leaf senescence depending on the environmental conditions, but the genetic mechanisms underlying this phenomenon remain poorly characterized. Notably, senescence-associated genes (SAGs) are upregulated in various plant species during senescence [6].

Abscisic acid (ABA), ethylene, jasmonic acid (JA), and salicylic acid (SA) signaling pathways can speed up senescence in older leaves [4,7,8]. ABA accelerates leaf aging by inducing the genes associated with chlorophyll breakdown and the genes linked to senescence [9,10]. JA and its derivatives are involved in leaf aging by inducing the expression of key chlorophyll degradation enzymes, like chlorophyllase [3,11]. JA also induces leaf senescence by upregulating JA biosynthesis genes [12]. The transcription factor WRKY53, associated with SA, stimulates the expression of ORESARA9 (*ORE9*), SENESCENCE-ASSOCIATED GENE12 (*SAG12*), and CATALASE1, 2, and 3 (*CAT1, 2,* and *3*), which are involved in advancing leaf senescence [13].

Several biotic and abiotic factors, dark, disease, and water stress can induce leaf senescence. Various molecular systems are also involved; for example, transcription of WRKY transcription factor 22 (*WRKY22*) is stimulated during dark-induced senescence [14]. However, few mutants associated with leaf senescence and abiotic stresses have been identified. Studying such mutants could help to elucidate the leaf senescence mechanism [15,16]. Plant defenses against pathogens with a hypersensitive response are characterized by localized cell death at the infection site. Lesion mimic mutations, which cause cell death in the absence of pathogen, often show enhanced resistance against pathogen infection [17]. Studies of these mutants could increase our understanding of the relationship between leaf senescence and plant defenses.

Leaf color is an important morphological trait recognizable by eye that can act as a reliable marker for plant breeding. Combinations of phenotypes and genotypes need to be scrutinized to elucidate the genetics of leaf color traits and physically map leaf color genes. The aim of this work was to identify the novel lesion mimic mutant brown midrib leaf (*bml*) and understand its role in leaf aging and defense responses against bacterial pathogens in rice. *BML* encodes an APK1A protein kinase, a typical receptor-like cytoplasmic kinase (RLCK), encoded by LOC_Os05g02020 or *OsRLCK176*. The function of this gene was characterized on the basis of leaf color phenotypes in the segregating progeny of the cross between *bml* and wild-type (WT) parents. The mutation in LOC_Os05g02020 causing the *bml* phenotype was detected by map-based cloning. The current findings will help to elucidate the roles of this mutation in different hormone signaling pathways and defense responses.

## 2. Materials and Methods

### 2.1. Plant Materials andGrowth Conditions

The rice (*Orzya sativa* L.) mutant *bml*, generated from the ethylmethane sulfonate (EMS)-induced *indica* cultivar ‘Zhenong 34’, was isolated by visual observation of phenotypes with early leaf senescence in an M_2_ population. The F_2_ population used for the genetic analysis was generated by crossing *bml* with WT ‘Zhenong 34’ (10 crosses were done using 10 WT plants). For gene mapping, the F_2_ population was developed by crossing ‘Zhenong 34’ with a *Oryza sativa japonica* cultivar ‘Zhenogda104’. All plants were grown to maturity in the rice fields of Zhejiang University in Hangzhou, China (30°15′49″ N, 120°7′15″ E), during March–July 2016.

### 2.2. Gene Mapping

Gene mapping was carried out using the bulk segregant analysis (BSA) method to select linked markers and the candidate gene [18]. The NCBI database [19], Gramene [20], DNASTAR, and Primer 5 software were used to design new polymorphic primers (Table S1). The Database of the Rice Genome Annotation Project [21] was used to obtain the function of each candidate gene in the region. Sequencing was conducted using candidate genes for the mutant and WT, and the mutation site was confirmed using NCBI BLAST [22].

### 2.3. Phylogenic Analysis of BML

The predicted PKc-like super family domain (OsRLCK) protein sequences of rice were aligned against a wide range of monocot and dicot crop species using Clustal Omega [23]. A phylogenetic tree was constructed with the MEGA 6.06 software using the neighbor-joining (NJ) algorithm [24]. To consistently analyze tree topology, 1000 replications for bootstrap values were used with full removal mode.

### 2.4. Estimation of Chlorophyll Content and Photosynthetic and Chlorophyll Fluorescence Parameters

The flag leaves of *bml* and WT plants were used to measure chlorophyll a (Chl a), chlorophyll b (Chl b), and total chlorophyll contents [25]. Photosynthetic parameters (i.e., net photosynthetic rate (Pn); stomatal conductance (gs); transpiration rate (Tr); intracellular CO_2_ concentration (Ci)) were quantified using an LI-6400 transportable photosynthesis system (LI-COR, Lincoln, NE, USA). The measurements were taken between 9:00 and 11:30 a.m. on a single day at the reproductive stage of growth. Using an imaging pulse-amplitude-modulated fluorometer (IMAG-MAXI; Heinz Walz, Effeltrich, Germany), the chlorophyll fluorescence of flag leaves of both *bml* and WT was measured after 30 min of adjustment to dark, as reported previously [26]. The maximal quantum yield of photosystem II (PSII) (Fv/Fm) for *bml* and WT was then calculated.

### 2.5. Microscopic Structure Analysis and Histochemistry Assayss

Paraffin section analysis was conducted as previously reported by Alamin et al. [27]. Briefly, the leaves were soaked overnight in FAA (10% formaldehyde, 50% ethanol, and 5% glacial acetic acid) at 4 °C, thereafter dehydrated in a 50–100% ethanol series, and finally set in paraplast. Microtome sections (10 μm) were stained using safranin (Sangon Biotech, Shanghai, China) and fast green (Sangon Biotech) and examined under an Image Pro-Plus 6.0 light microscope (Sangon Biotech). For transmission electron microscopy (TEM), the middle part of the second leaf blade and midrib from *bml* and WT leaves were fixed in 2.5% glutaraldehyde, subsequently set in 1% osmium tetraoxide (Sangon Biotech), desiccated in a graded acetone progression, finally permeated in Epox 812, and fixed. After staining with methylene blue (Sangon Biotech), ultra-thin pieces were sliced and stained with uranyl acetate with lead citrate (Sangon Biotech). The sections were observed using a transmission electron microscope (HITACHI, H-600IV, Tokyo, Japan).

Histochemical cell death experiments and assays of reactive oxygen species (ROS) aggregation, were carried out as previously described by Zhou et al. [28]. The samples were flooded with a lactic acid–phenol–trypan blue (Sangon Biotech) solution (2.5 mg/mL trypan blue, 25% (*w*/*v*) lactic acid, 23% water-soaked phenol) at 70 °C, along with 25% glycerol, then permeated for 20 min, warmed in hot water for 2 min, chilled for 1.5 h to allow trypan blue staining, and destained using chloral hydrate (2.5 g/mL). To measure the levels of superoxide (O_2_^−^), the leaf samples were absorbed in 0.5 mg/mL nitro blue tetrazolium (NBT) (Sangon Biotech) in 10 mM potassium phosphate buffer (pH 7.8) in the dark for 16h. To measure hydrogen peroxide (H_2_O_2_), the leaf samples were steeped in 1mg/mL 3,3′-diaminobenzidine (DAB) (Sangon Biotech) and 10 mM 2-(N-morpholino) methanesulfonic acid (MES, pH 6.5) (Sangon Biotech) in the dark for 18 h. Leaf chlorophyll was removed by treating with 75% ethanol in hot water for 10 min, then placing in absolute ethanol.

### 2.6. Dark-Induced Treatment

Naturally grown, healthy (green and spotless) flag leaves were wrapped in aluminum foil to maintain the dark conditions in the rice field for 10 days during heading. Five flag leaves from the mutant and five from the WT were randomly chosen for the treatment, as described previously by Sun et al. [29].

### 2.7. Measurement of Antioxidant Enzyme Activity

To measure the activities of the antioxidant enzymes superoxide dismutase (SOD), catalase (CAT), and peroxidase (POD), and the malondialdehyde (MAD) content, 0.5 g samples of leaf were used for each treatment. The samples were homogenized in 8 mL of 50 mM potassium phosphate buffer (pH 7.8) by using a chilled mortar and pestle to maintain an ice-cold environment, as described by Ahmed et al. [30].

### 2.8. Hormone Measurement

Different phytohormones from the tips of the second leaves of *bml* and WT plants were extracted and measured 10 days after flowering [31]. The leaves were frozen and crushed in liquid nitrogen, and endogenous indole-3-acetic acid (IAA), ABA, JA, and SA were quantified using three replicated samples from independent plants with ABA, IAA, JA, and SA ELISA kits (MLBio ELISA Kit producers, Shanghai, China), according to the manufacturer’s instructions. A multilevel calibration graph was made with internal standards.

### 2.9. Inoculation with Bacterial Blight Pathogen

New, fully expanded leaves of six independent *bml* and WT plants at the tillering stage were inoculated with Guangzhou-C (Gz-C) suspensions of *Xanthomonas oryzae* pv. *oryzae* bacteria (absorbance at 600 nm was 0.5) using the clipped leaf technique. Disease progress, measured as damage lengths in the leaves, was scored 21 days after inoculation [32].

### 2.10. RNA Extraction and Quantitative Reverse Transcription Polymerase Chain ReactionAnalysis

Ex Taq II was used for reverse transcription polymerase chain reaction (qRT-PCR) as described in the Takara instruction leaflet (Takara, Tokyo, Japan). The primers for qRT-PCR are listed in Table S2. PCR was executed using the following profile: denaturation at 95 °C for 30 s, then 40 cycles of denaturation at 95 °C for 5 s, annealing at 55 °C for 20 s, extension at 72 °C for 10 s. Rice actin was used as a reference gene [33].

### 2.11. Statistical Analysis

All data are shown as means ± standard deviation (SD) of five replicates. The statistical software package SPSS (version 20) (IBM corporation, Armonk, North Castle, NY, USA) was used for data analysis. One-way analysis of variance and subsequently a Tukey’s test were performed for pair-wise statistical significance difference of means. The graphs were prepared using Origin Pro version 8.0 (Origin LabCorporation, Wellesley Hills, MA, USA). A Χ^2^-test was used to detect the separation ratio of the *bml* mutant in the segregating F_2_ population.

## 3. Results

### 3.1. Effect of the bml Mutation on Phenotype

**Figure 1:**
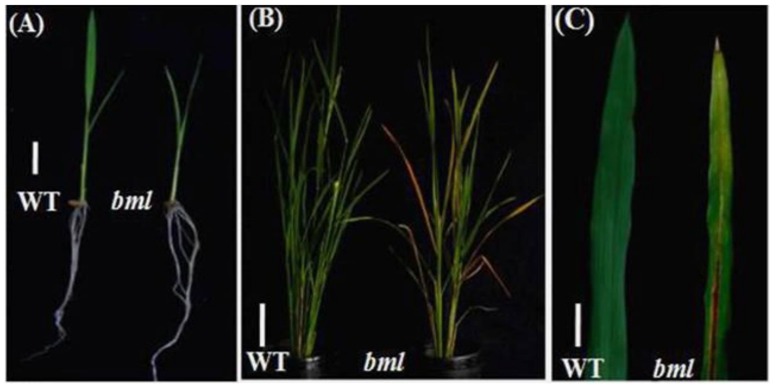
Phenotypic characterization of brown midrib leaf (*bml*) mutant and wild-type (WT) rice plants. (**A**) Morphologically similar phenotypes observed in *bml* and WT at the seedling stage. Bar = 2 cm. (**B**) Phenotypes of *bml* and WT at the heading stage. Bar = 10 cm. (**C**) Enlarged view of part of the second leaves from the top of the plant shown in (B). Bar = 5 cm.

Plants with the *bml* mutation were identified from a screen of mutants in the M_2_ generation by the appearance of the early senescence phenotype compared with WT. The *bml* mutants did not display a browning phenotype at the seedling stage (Figure 1A). However, compared with WT, whose leaves stayed green, the lower leaves of *bml* began to turn yellow with progressive senescence at the heading stage under natural conditions (Figure 1B). The midrib of the *bml* leaves started to brown, and lesion-like spots appeared on the leaf blade, gradually covering the whole leaf, which became senescent (Figure 1C). In addition, the *bml* plants grew very slowly, resulting in small plants with a significantly reduced number of tillers per plant, panicle length, and seed-setting compared to WT (Figure S1). Reduced numbers of grains in the panicles, lower 1000-grain weight, and smaller seed size (grain length and width) were also recorded in the mutant (Figure S1).

A microscopic observation of cross sections of the leaves of *bml* and WT (Figure 1C) revealed that the leaves of *bml* plants were narrower than those of WT. Obvious variations between *bml* and WT were seen in terms of the number, size, and shape of bulliform cells (Figure S2A,B) that were small and shrunken in *bml* but fully expanded in WT (Figure S2C,D). The narrower *bml* leaves might be caused by the altered morphology of the bulliform cells [34]. In addition, a light-induced lesion mimic phenotype was observed in *bml* leaves grown in natural light conditions, whereas these lesions were absent in WT and in dark-induced leaves of *bml* or WT (Figure S3).

### 3.2. Genetic Analysis, Map-Based Cloning, and Expression Pattern Analysis of the BML Gene

**Figure 2:**
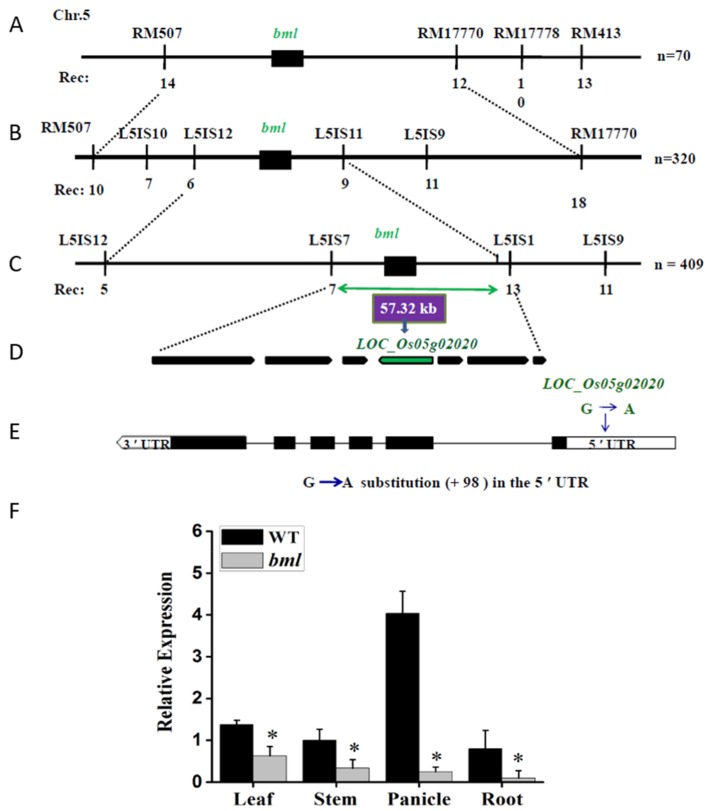
Map-based cloning of the *BML* locus. (**A**) Primary mapping between two markers, RM507 and RM17770 (linked to *BML*), on the short arm of chromosome 5. Seventy F_2_ individuals of the mutant were used; the corresponding number of recombinants is listed (below the vertical lines). (**B**) Using 320 F_2_*BML* mutant individuals, *BML* was primarily mapped to a region between the markers L5IS12 and L5IS11. (**C**) Finally, using 409 F_2_ mutant individuals, *BML* was fine-mapped to a 57.32 kb interval region between the markers L5IS7 and L5IS11. (**D**) Seven genes were annotated in the 57.32 kb candidate region; LOC_Os05g02020 was presumed to be *BML*. (**E**) The structure of the *BML* gene and the locus of the mutation are marked. Black boxes indicate exons, and lines indicate introns. (**F**) Expression patterns of *BML* in rice leaf, stem, panicle, and root at the heading stage. The rice actin gene was used as a reference. All data are shown as means ± standard deviation (SD) of five replicates; * *p* < 0.05 by Tukey’s method.

The F_1_ generation of a cross between *bml* and WT rice plants had a similar phenotype to that of the WT. In the F_2_ generation, 209 plants had the WT phenotype, and 63 plants had the mutant phenotype, fitting a segregation ratio of 3:1 (*χ*^2^= 0.490 < *χ*^2^_0.05_ = 3.84) and confirming the presence of a single copy of a recessive *bml* allele. To identify the chromosomal location of *BML*, an F_2_ population was generated from a cross between *bml* and ‘Zhenongda 104’. Map-based cloning revealed the locus on the short arm of chromosome 5 between the simple-sequence repeat (SSR) markers RM507 and RM17770 (Figure 2A). Using the genome sequences of *indica* and *japonica* as references, 10 new InDel markers were designed to construct a high-resolution genetic and physical map. Using 320 individuals of the F_2_, the *BML* gene was mapped between the markers L5IS12 and L5IS11 (Figure 2B). *BML* was fine-mapped to a 57.32 kb region between InDel markers L5IS7 and L5IS11, using 409 individuals of the mutant from the F_2_ of *bml*/‘Zhenongda 104’ (Figure 2C). According to the Rice Genome Annotation Project [21] database, seven putative genes were expected in the 57.32 kb gap, encoding an NAD-dependent epimerase/dehydratase family protein (homolog LOC_Os05g01970), a DEAD-box ATP-dependent RNA helicase (LOC_Os05g01990), a rab5-interacting-like protein (LOC_Os05g01994), protein kinase APK1A, a chloroplast precursor (LOC_Os05g02020), an OB-fold nucleic acid-binding domain protein (LOC_Os05g02030), RPA1C, a single-stranded DNA binding complex subunit 1 (LOC_Os05g02040), and the mitochondrial import inner membrane translocase subunit Tim (LOC_Os05g02050) (Figure 2D). Genomic DNA was used to sequence the genes from *bml* and WT plants. In terms of the sequences of these genes, no differences were found between *bml* and WT, except for the sequence of LOC_Os05g02020. Therefore, LOC_Os05g02020 was chosen as the candidate gene for further detailed sequence analysis. Detailed DNA sequencing analysis of the candidate gene showed a 1bp substitution (G to A) in the 5′ untranslated region (UTR, +98) of *bml* (Figure 2D,E). RECEPTOR-LIKE CYTOPLASMIC KINASE 176 (*OsRLCKl76*) or Os05g0110900 (RAP-DB locus) was situated on the reverse DNA strand of chromosome 5 (Chromosome 5: 580,488–577,144) [20,21], which encodes an APK1A protein kinase. The genomic DNA and cDNA sequences of *BML* were 3345 bp and 1188 bp, respectively, and the 5′ and 3′ UTR were 643 bp and 355 bp, respectively. This gene comprised six exons and five introns with 395 amino acid residues. Therefore, LOC_Os05g02020 was determined to be the gene most likely to cause the *bml* mutation.

Quantitative RT-PCR using complementary DNA (cDNA) from leaf, stem, panicle, and root tissues at the heading stage revealed the expression of *BML* to be ubiquitous in these organs (Figure 2F). However, the expression of this gene was lower in the leaf, stem, panicle, and root tissues of *bml* than in the corresponding elements of WT (the expression was decreased by 54%, 67%, 94%, and 88% in the leaf, stem, panicle, and root, respectively). This reduced expression might be caused by the substitution of G to A at the 5′ UTR, affecting the transcriptional level of *bml* and leading to a phenotypic change in the rice plant.

### 3.3. Phylogenetic Analysis and Domain Location

In plants, RLCKs belong to the receptor-like kinase (RLK) superfamily, have homology with RLKs in their kinase domain but lack the transmembrane domain. Fourteen major types of RLCK domain organizations have been reported to date [35], although this phylogenetic analysis was done to understand the relatedness and evolutionary relationships between plant species containing genes with PKc-like conserved domains. Using 43 full-length protein sequences of PKc-like domains, including five from the *Oryza* genus, one from *Arabidopsis*, and others from a variety of crop and fruit species, a phylogenetic tree was constructed to understand the evolutionary relationships between PKc-like family proteins in rice. The PKc-like family is divided into four well-conserved clades (I–IV) with significant bootstrap support (Figure S4). The phylogenetic and domain analysis revealed that the PKc-like domains of RLCKs are divergent in different plant species but conserved in cultivated rice. The selected gene locus LOC_Os05g02020 from *Oryza sativa japonica*, and the gene reported here, *OsRLCK176*, from *Oryza sativa indica*, fall in the same group, indicating their common ancestral origin.

### 3.4. Chlorophyll Content, Photosynthesis, and Chlorophyll Fluorescence in bml

**Figure 3:**
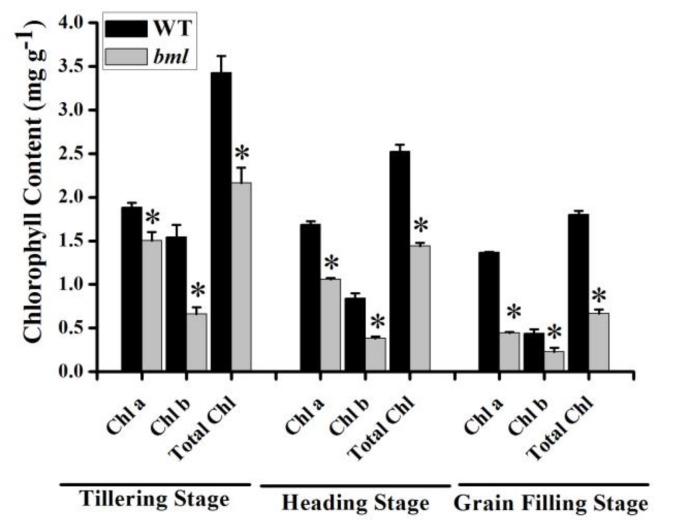
Photosynthetic pigment content analysis. Contents of the photosynthetic pigments in the leaves during the tillering, heading, and grain-filling stages in *bml* and WT. Chl: chlorophyll; Chl a: chlorophyll a; Chl b: chlorophyll b. All data are shown as means ± SD of five replicates; * *p* < 0.05 by Tukey’s method.

To determine any effects of the mutation of the *BML* locus on chlorophyll content and photosynthesis, the contents of the photosynthetic pigments in the flag leaves of *bml* and WT were investigated at different stages (Figure 3). In *bml*, photosynthetic Chl a, Chl b, and total chlorophyll contents were significantly decreased at the tillering, heading, and grain-filling stages compared to WT. However, the Chl a/Chl b ratio was significantly higher in *bml* than in WT at the tillering stage, suggesting smaller light-harvesting antennae. By contrast, the Chl a/Chl b ratio was 2.76 and 2.01 in *bml* and WT, respectively, at heading. However, in *bml*, the ratio dropped at the grain-filling stage. This suggests damage to the photosystems (Table S3).

The photosynthetic characteristics of *bml* and WT plants were observed at the peak tillering and reproductive stages by measuring gas exchange in the flag leaves (Table S4). In *bml*, photosynthetic rate (Pn), stomatal conductance (Gs), intercellular CO_2_ concentration (Ci), and transpiration rate (Tr) were significantly lower than in WT. The photosynthetic parameters were also decreased in both *bml* and WT at the grain-filling and reproductive stages, with significant differences in Pn, Gs, Ci and Tr between *bml* and WT (Table S4).

To determine whether the photosynthetic apparatus was functional in *bml* plants, the maximal efficiency of PSII (Fv/Fm) was compared in *bml* and WT plants at the reproductive stage using a pulse-amplitude modulation (PAM) chlorophyll fluorometer. The Fv/Fm (maximal quantum yield of PSII) in *bml* was decreased by 17.2% compared to WT (Figure S5).

### 3.5. Ultrastructure Changes in Mesophyll Cells and Leaf Chloroplasts

**Figure 4:**
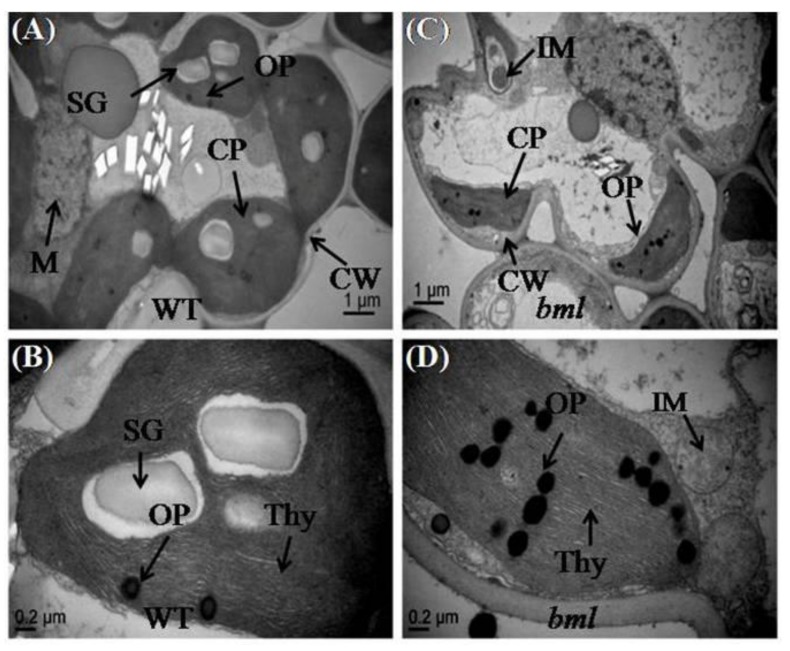
Ultrastructure of chloroplasts in the middle part of the second leaf blade from the top of *bml* mutant rice plants. (**A**,**C**) Magnified view of chloroplasts in mesophyll cells of *bml* and WT plants at the heading stage. Bar = 1 μm. (**B**,**D**) Transmission electron microscope images (TEM) of chloroplasts and thylakoid membranes in the mesophyll cells of *bml* mutants and WT rice plants. Bar = 0.2 μm. CP: chloroplast; CW: cell wall; Thy: thylakoids; OP: osmophilic plastoglobuli; SG: Starch Granule; M: mitochondria; IM: immature mitochondria.

The observation of the ultrastructure of whole mesophyll cells and chloroplasts in the middle part of the second leaf from the top of both *bml* and WT plants at the heading stage, revealed various alterations in cell size and chloroplast structure (Figure 4A–D). In *bml* plants, mesophyll cells and chloroplasts had irregular shapes and sizes; there were no starch granule, immature mitochondria (M), obvious cell walls (CW), and larger vacuoles. Moreover, the chloroplast lamellae were collapsed and the number of disintegrated osmophilic plastoglobuli and shrunken chloroplasts with incomplete thylakoids was increased (Figure 4B,D). In WT plants, TEM of the middle part of the leaf blade revealed the presence of nearly equal-sized, regularly shaped, and properly arranged chloroplasts with larger starch granule, well developed mitochondria (M) and thylakoids (Thy), distinct cell walls, and fewer osmophilic plastoglobuli in leaf mesophyll cells (Figure 4A,C).

**Figure 5:**
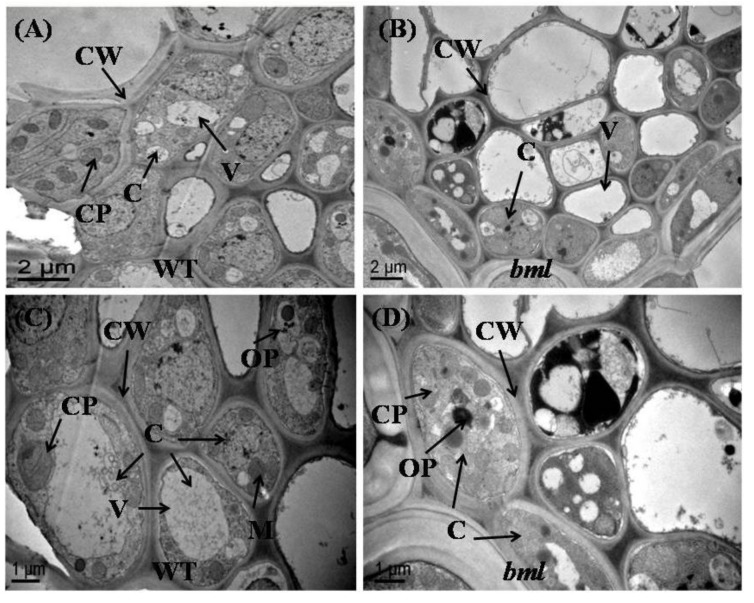
Ultrastructure of mesophyll cells in the middle part of the midrib of the second leaf from the top of *bml* mutant and WT rice plants at the heading stage. (**A**,**B**) TEM images of whole leaf mesophyll cells of *bml* and WT. Bar = 2 μm. (**C**,**D**) relatively low magnification view of mesophyll cells of *bml* and WT. Bar = 1 μm. C: cell; CW: cell wall; V: vacuole; CP: chloroplast; OP: osmophilic plastoglobuli; M: mitochondria.

To ascertain whether the cell structure of the midrib of *bml* was altered, the ultrastructure of brown sections of the middle part of *bml* and WT plant’s midrib was observed under TEM. The cells of WT leaves contained mature, organized cell components with a well-organized cell wall (Figure 5A,C). In contrast, the *bml* cells had abnormal, immature, disorganized cell organelles, and an increased number of osmophilic plastoglobuli (Figure 5B,D).

### 3.6. Leaf Senescence-Associated Gene Expression in bml

**Figure 6:**
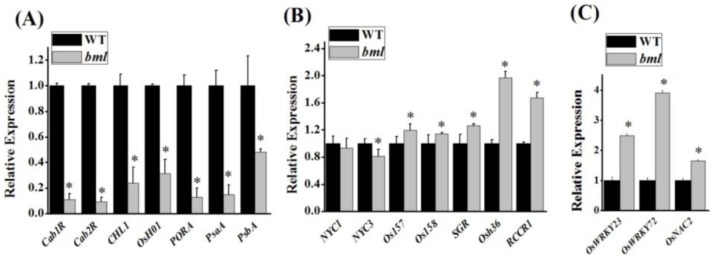
Expression of genes associated with leaf senescence in *bml* using quantitative reverse transcription polymerase chain reaction (qRT-PCR). Expression analysis of (**A**) photosynthesis-associated genes, (**B**) senescence-associated genes, and (**C**) senescence-associated transcription factors of *bml* mutants and WT rice plants at the heading stage. The expression levels are relative to rice *Actin*. All data are shown as means ± SD of five replicates; * *p* < 0.05 by Tukey’s method.

An expression profiling was conducted to understand the effects of the *bml* mutation on the expression of other leaf senescence-associated genes. The expression patterns of chlorophyll degradation and leaf senescence-related genes at the early heading stage revealed that the expression of the photosynthesis-related genes *CAB1R*, *CAB2R*, *CHL1*, *OsH01*, *PORA*, *PSAA*, and *PSBA* was significantly decreased in *bml* (Figure 6A) compared to WT. However, the expression of the senescence-associated genes *Os157*, *Os158*, *SGR*, *Osh36*, and *RCCRI* was significantly increased. *NYC3* and*NYC1* were downregulated in *bml*, but there was a non-significant difference in the transcript abundance of *NYC1* between *bml* and WT (Figure 6B). The expression of the senescence-associated transcription factor genes *OsWRKY23*, *OsWRKY72*, and *OsNAC2* was higher in *bml* at the early heading stage, corresponding to early leaf senescence (Figure 6C). These findings reveal the unusual decline of light-harvesting chlorophyll protein complexes within the thylakoid membranes due to untimely leaf senescence. In addition, some upregulated genes showed differential expression indicating that the *bml* mutation might not affect all of the upregulated genes in a similar manner.

### 3.7. Activities of Antioxidant Enzymes and Reactive Oxygen Speciesin bml

**Figure 7:**
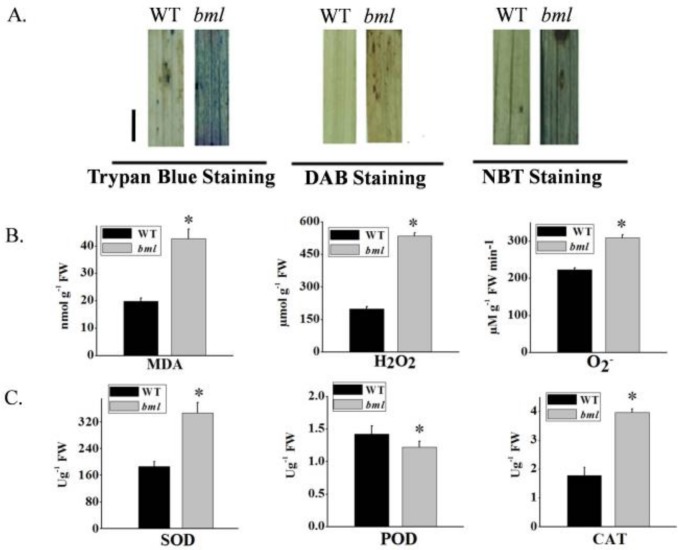
Histological staining and measurement of the activities of enzymes involved in scavenging and generating reactive oxygen species. (**A**) Analysis of histologically stained *bml* and WT rice leaves at the onset of heading, bar = 1 cm (**B**) Contents of malondialdehyde (MDA), hydrogen peroxide (H_2_O_2_), and superoxide anions (O_2_^−^). (**C**) Contents of the enzymes superoxide dismutase (SOD), ascorbate peroxidase (POD), and catalase (CAT) in *bml* and WT at the onset of heading. All data are shown asmeans ± SD of five replicates; * *p* < 0.05 by Tukey’s method. DAB: diaminobenzidine; NBT: nitro blue tetrazolium.

To measure the activities of antioxidant enzymes and ROS in *bml*, the leaves were stained with trypan blue dye. The leaves of *bml* showed blue-colored spots, whereas no staining was observed in WT leaves, indicating severe cell necrosis in *bml* (Figure 7A). When stained with diaminobenzidine (DAB), *bml* leaves presented brown-stained spots, whereas no staining was detected in WT leaves (Figure 7A). In *bml* leaves stained with nitro blue tetrazolium (NBT), blue deposits were observed, indicating superoxide (O_2_^−^) accumulation. The leaves of *bml* at the tillering stage also accumulated much more hydrogen peroxide and superoxide radicals than WT leaves (Figure 7B). Malondialdehyde (MDA) is produced as a result of membrane lipid peroxidation; its accumulation can reveal membrane injury, ultimately reflecting the level of cellular damage. Compared to WT, MDA levels were significantly increased in *bml* leaves at the same stages of growth (Figure 7B). The activities of the oxidative stress-related enzymes CAT and SOD were significantly higher in *bml* than in WT, and POD activity was significantly lower (Figure 7C).

### 3.8. Disease Response and Upregulation of Pathogenesis-Related Marker Genes

**Figure 8:**
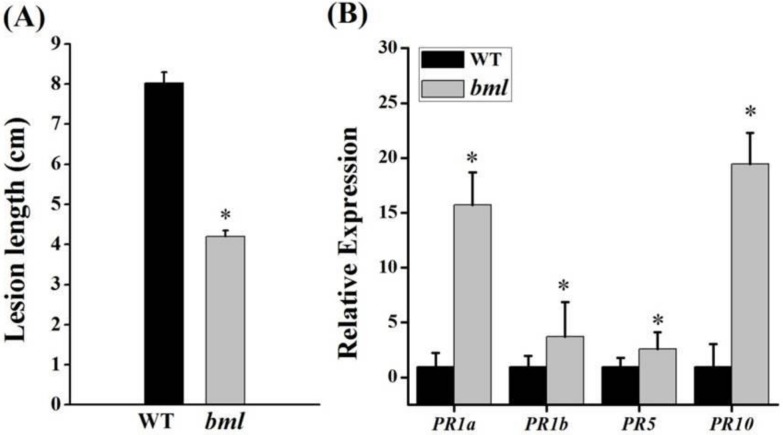
Bacterial blight resistance and expression of resistance-related genes. (**A**) The lesion lengths were measured after inoculating *bml* and WT plant leaves with the bacterial blight pathogen *Xanthomonas oryzae* pv. *oryzae* strain Gz-C. The bars show means ± SD of 10 replicates. (**B**) Expression of pathogenesis-related (PR) marker genes at the tillering stage. All data are shown as means ± SD of five replicates; * *p* < 0.05 by Tukey’s method.

To assess the disease response and expression of four pathogenesis-related (*PR*) genes, *bml* and WT plants were inoculated with *X. oryzae* pv. *oryzae* (*Xoo*)bacteria and scored for disease after 21 days. The appearance of necrotic spots in *bml* suggested the activation of the hypersensitive response (HR), a resilience system in plants. In *bml* plants, the lesion length (~4 cm) was shorter than in WT (~8 cm), indicating enhanced *Xoo* resistance in the mutant (Figure 8A). The expression of four pathogenesis-related (*PR*) marker genes (*PRIa*, *PRlb*, *PR5*, and *PR10*) was significantly upregulated, and, among them, two of the defense genes (*PRIa* and *PR10*) were highly upregulated, i.e., over 10-fold, in *bml* compared with WT (Figure 8B), indicating an association between the *bml* mutant and positive defense responses in rice.

### 3.9. Measurements of Phytohormones and Signaling Pathway Gene Expression

**Figure 9:**
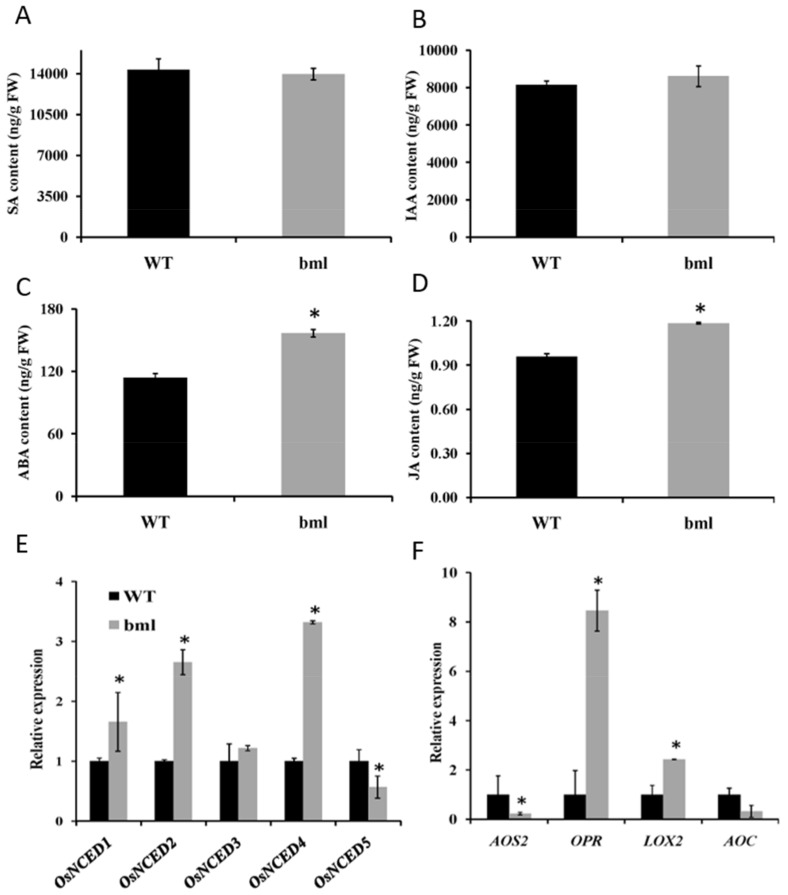
Levels of different phytohormones associated with senescence in *bml* and wild-type (WT) plants. Contents of (**A**) salicylic acid (SA), (**B**) indole-3-acetic acid (IAA), (**C**) abscisic acid (ABA) and (**D**) jasmonic acid (JA), in the second leaves from the top of *bml* and WT plants at the heading stage. Expression analysis of (**E**)ABA biosynthetic genes and (**F**) JA biosynthetic genes using the second leaf from the top of *bml* and WT plants at the heading stage. All data are shown as means ± SD of five replicates; * *p* < 0.05 by Tukey’s method.

To determine whether the *bml* mutation affects hormone content and the expression of signaling pathway genes, phytohormones SA, IAA, ABA, and JA contents were measured in *bml* and WT leaves. No significant difference was found between *bml* and WT plants in SA or IAA contents (Figure 9A,B); however, JA and ABA levels were significantly higher in *bml* than in WT (Figure 9C,D), which might enhance senescence in *bml*. The expression of the ABA synthesis genes *OsNCED1*, *OsNCED2*, and *OsNCED4* were significantly higher in *bml* than in WT, while *OsNCED5* was significantly downregulated in *bml* (Figure 9E). In addition, the JA synthesis-related genes *LOX2* and *OPR7* were upregulated, but *AOS2* was significantly downregulated in *bml* compared to WT (Figure 9F).

## 4. Discussion

Early leaf senescence adversely affects growth and rice yields because of its detrimental effects on photosynthesis. The role of early leaf senescence as an important defense response in rice and its molecular regulation remain poorly understood. We isolated and characterized a rice mutant, *bml*, which has an altered senescence phenotype, to understand the genetic importance of leaf senescence. Compared with WT, *bml* plants demonstrated early leaf senescence and had slower growth and lower yields. These traits might be attributed to detrimental effects on the efficiency of the photosynthetic system [31,36]. Leaf browning initiated at the midrib and spread across the whole leaf, which gradually became senescent. Anatomical (microscopic observation), physiological, and biochemical analysis confirmed that leaf senescence occurred because of reduced chlorophyll and photosynthetic parameters and chloroplast degradation, similar to observations reported in a previous study [1,3,31].

Environmental conditions might also induce the lesion mimic phenotype [37]. It is noteworthy that photorespiration is important in protecting photosynthetic organs from damage due to excessive absorption of light energy [38]. In *bml*, lesions were induced by light in leaves with reduced photosynthetic capability, indicating that light might trigger accelerated oxidative damage in *bml* leaves. Early leaf senescence occurred in *bml*, as confirmed by the observation of chloroplast degradation (Figure 4C), upregulation of aging-related transcription factors (*OsWRKY*2, *OsWRKY72*, and *OsNAC2*) and senescence-associated genes, and downregulation of photosynthesis-related genes (Figure 6A–C). Therefore, the tissues in *bml* seemed to be susceptible to oxidative damage and initiated senescence quicker than WT tissues.

Chloroplasts in the *bml* leaves contained unusual starch granules, indicating irregular starch metabolism and a reduced ability to deliver energy for development. Furthermore, *bml* chloroplasts had a greater number and larger size of osmophilic bodies without granule lamellae. These damaged, abnormal chloroplasts are likely to be responsible for premature leaf senescence [39].

*BML* was expressed in leaf, stem, root, and panicle tissues in both *bml* and WT plants [40]. A point mutation in the 5′UTRinduced alterations in the expression of *BML* in different tissues of rice [41]. In *bml* plants, a G to A substitution in the 5′UTR of *BML* might cause the significant reduction of transcript abundance compared to WT in the collected tissues. Previous studies analyzing different parts of *OsRLCK176Ri* transgenic rice plants found similar expression patterns of *OsRLCK176* in different rice tissues and increased resistance to *Xoo* [40]. This point mutation affected the plastid, where JA biosynthesis is initiated. In the *bml* mutant, higher levels of JA werefound, which might be related to defense responses against bacteria and the detrimental effect on the development of different organs in rice. Exogenous methyl jasmonate (MeJA) applications on rice seedlings reduced the growth of roots shoots, panicle, spikelets etc.[42], and the overproduction of JA induced alesion-mimic phenotype on the leaves and causedtheir senescence [43]. Here, we also reported similar *OsRLCK176* expression patterns and increased *Xoo* resistance in *bml*, as well as similar phenotypic changes to those reported by Ao et al. [44], in which three independent transgenic rice lines carrying an *OsRLCK176* RNA interference (RNAi) mutation were evaluated.

In the present study, cloning the *BML* gene revealed that it encodes an APK1A protein kinase, a typical RLCK represented by LOC_Os05g02020 or *OsRLCK176* (RAP-DB locus). As found in a previous study, phylogenetic and domain analyses revealed that the PKc-like domains of *RLCK* genes are divergent among different plant species but remain conserved in cultivated rice species [35]. A previous study noted that enzymes involved in antioxidant systems are expressed in lesion mimic mutants [45]. In *bml*, early leaf senescence correlated with the physiological indicators of decreased pigment content, increased MDA content, and increased SOD activity. MDA content was considerably higher, while chlorophyll content and photosynthetic rates were lower from the peak tillering to the grain-filling stage because of premature leaf aging. Here, large numbers of hydrogen peroxide and superoxide anions accumulated in *bml*, which thereafter induced CAT activity and greatly reduced POD activity. Thus, the accumulation of ROS in the mutant can most likely be attributed to an over production of hydrogen peroxide and a damaged radical scavenging pathway.

In *Arabidopsis*, overexpression of *OsWRKY23* affected senescence and resistance, increasing the expression of *PR* genes and improving immunity against the bacterial pathogen *Pseudomonas syringae* [46]. The regulation of RLCK proteins can be involved in defense signaling. At the onset of the tillering stage, the *bml* mutant displayed disease-like lesions in the absence of pathogen attack. In turn, defense-response genes were activated, and bacterial disease resistance was enhanced, suggesting that *OsRLCK176* positively regulates the defense response. This is consistent with published data showing that *OsRLCLK176* is important in broad-spectrum bacterial blight resistance [40]. However, reduced *OsRLCK176* expression results in a leaf senescence phenotype, possibly because defense and leaf senescence might be tightly coupled, and thus we found that four *PR* genes were upregulated in the *bml* mutant (Figure 8B). In the plant disease resistance system, there are two main signaling pathways, i.e., one involving JA, and one involving SA [47]. Since the expression of both *PR1a* and *PR1b* were increased in the mutant, *bml* might be involved in both JA and SA hormone signaling pathways. Moreover, CLH is responsible for chlorophyllide development upon the degradation of leaf cells and ultimately causes leaf senescence [3]. We suppose that chlorophyllide formation might form part of the defense response against pest or pathogen attack.

In our study, leaf senescence was primarily associated with increased levels of phytohormones such as ABA, JA, and SA, which are heavily involved in responses to different stresses [48,49]. ABA is a key plant hormone that mediates environmental stresses. Previous work demonstrated that ABA contents are higher in senescing leaves, and exogenous ABA induced the expression of several SAGs [29]. This was consistent with the leaf aging phenotype in *bml*. The expression levels of the genes encoding 9-cis-epoxycarotenoid dioxygenase (NCED), the key enzyme involved in ABA biosynthesis, were increased along with increased ABA in senescence leaves [48,50].

Four ABA biosynthesis-associated genes were significantly upregulated in *bml*. In addition, JA might be induced to produce chlorophyllase—the main enzyme of chlorophyll degradation [3,11]—promoting the loss of chlorophyll in the leaves. JA causes the expression of several senescence-associated genes to accelerate senescence [51,52]. Transcripts of genes encoding enzymes in the JA biosynthesis pathway increased sharply in *bml*, along with the expression levels of *LOX2* and *OPR7* (Figure 9F). An accelerated leaf senescence in *bml* plants might therefore have caused a subsequent increase in ABA and JA levels.

ROS are also important in early leaf senescence [53]. ABA and ROS signaling induce the expression of aging-coupled transcription factors [7,12,54]. Manipulation of *BML* might provide a new line of inquiry for genetic engineering to develop new resistant rice lines. Likewise, genetic evidence suggests that ROS do not activate senescence but act as a signal to stimulate the genes that regulate the events involved in cell death [55]. Mutation of *BML* accelerated leaf senescence associated with increased ROS and ABA signaling; however, the connection between JA, ABA, ROS, and leaf senescence remains to be established.

## 5. Conclusions

A rice mutant, *bml*, was characterized by its phenotype consisting of senescent leaves with a brown midrib at the onset of heading. The mutant had abnormal cells, degraded chloroplasts, and dramatically reduced chlorophyll contents. A genetic analysis confirmed that the *bml* trait is controlled by a single recessive nuclear gene, the result of a 1 bp substitution (G to A) in the 5′-UTR (+98) of *BML*. This gene was fine-mapped to a 57.32 kb interval between the L5IS7 and L5IS11 *InDel* markers located on the short arm of chromosome 5. ABA and JA contents were increased in *bml*, and some ABA and JA biosynthesis genes were upregulated. *bml* plants were more resistant to *X. oryzae* pv. *oryzae* than WT plants. In the *bml* mutant, higher level of JA and high upregulation of two defense genes (*PRIa* and *PR10*) seem to confer resistance against bacterial pathogens. These findings deepen our understanding of the mechanisms of leaf senescence associated with ROS and hormone signaling pathways in rice.

## Supplementary Materials

The following are available online at http://www.mdpi.com/2073-4425/9/4/203/s1, Figure S1: Comparison of the agronomic traits between *bml* and WT. The bars symbolized standard deviations of three independent measurements. The asterisks imply a significant difference at *p* < 0.05 using Tukey’s method. Figure S2: Histo-cytological micro-structure of the flag leaf and main vein. (A,B) Cross sections of the flag leaf blades in *bml* and WT at the heading stage, respectively. Bar = 100 μm. (C,D) The amplification of the main vein in *bml* and WT, respectively. Bar = 200 μm. BC—bulliform cells, LV—large vascular bundle, SV—small vascular bundle. Figure S3: Leaves of *bml* and WT at the tillering stage on the right panel were covered with aluminum foil for 10 days to see the effect of dark and sunlight on the appearance of brown leaf spots. Figure S4: Phylogenetic relationship using 43 full-length protein sequences of PKc-like domains of *RLCK* genes, which included four genes from *Oryza sativa*, one from *Arabidopsis*, and the rest from a wide range of different crop and fruit species, to get insights into the evolutionary relationships among rice PKc-like domains by the neighbor-joining (NJ) method with MEGA 6.0. The numbers on the branches indicate bootstrap support values from 1000 replications. The tree was divided into four clades according to the bootstrap support values and evolutionary distances. The accession numbers of different sequences with their species name are presented in abbreviated form at the end of each accession. Figure S5: (A) Comparison of flag leaf phenotype and the maximum efficiency of photosystem II photochemistry (Fv/Fm) image. (B) The Fv/Fm value of *bml* compared with that of WT. The asterisks indicate statistically significant differences at *p* < 0.05 using Tukey’s method.
