# Supplementary material for: The Brown Midrib Leaf (bml) Mutation in Rice (Oryza sativa L.) Causes Premature Leaf Senescence and the Induction of Defense Responses
Source: Genes (Basel). 2018 Apr 9;9(4):203. doi: 10.3390/genes9040203 (PMC5924545; doi:10.3390/genes9040203)
Supplement: Supplementary file 1 [file genes-09-00203-s001.zip › Supplementary materials/Supplementary Tables.docx]

**Table S1** List of the primers used in this study for gene mapping.

| **Name** | **Forward primer (5´–3´)** | **Reverse primer (5´–3´)** |
| --- | --- | --- |
| RM507 | AACTGCCATCTCTGAAACTCTGC | CATCTCACTTCAGAAGGATCATAGCC |
| RM17770 | GATCAGTGAGAGTGAGGTGGAAGG | AGGATGGATACATGGAAGGAAGG |
| RM17787 | GGGTACACGTGATGGCTTGAGAGC | ACCGGGAGGAGATCATGCTAGACC |
| RM413 | CCAATCTTGTCTTCCGGATCTTGC | AGATAGCCATGGGCGATTCTTGG |
| L5IS7 | CATTTCCTGCCTTGAGTG | CCCCATCGAAGAATTGTTAT |
| L5IS9 | ATCATTGCGATTGTTCTAG | TTGGATGGTAAACCCTCTT |
| L5IS10 | TCCTGCCAGACAAGAAGTT | GCTCATCACTGGAACCCAC |
| L5IS11 | CGGGTCTGGAGCTTGTCGGCGA | CCTTCTTGCCGAGGGTTCGCC |
| L5IS12 | TCAGTGTTAGGACCACCAGC | ACTTCATCCCTGACATAGCG |
| L101 | AAGTATGAATGGCAAACAGTGG | GAGATGCTATCAGGACGACG |
| L102 | GCTCTTGGTGGTCAGATGGC | GTGCTTGGTGAGGGAGGCTT |
| L103 | CGTCTTCTCATCGATCCACCCT | TACAAATCTCTTGCTCCCCTCC |
| L104 | TCGGAGGTAGTAGAGCCCAA | GCAGCCAAGCAAAACAGAAAAA |
| L103 | CGTCTTCTCATCGATCCACCCT | TACAAATCTCTTGCTCCCCTCC |
| Sub-cellular localization by GFP | CGGGGGACGAGCTCGGTACCATGGGGAATTGCTGGGGC | CAGTGAAAAGTTCTTCTCCTTTACTCATTCTAGAAACCAGCCTCGCATTTGC |

**Table S2** List of the primers used in this study for qRT PCR.

| Name | **Forward primer (5´–3´)** | **Reverse primer (5´–3´)** |
| --- | --- | --- |
| *Actin* | GTGGTCGCCCCTCCTGAAAG | GGCTTAGCATTCTTGGGTCCG |
| *LQRT* | GAATGGCTGGCTGAAGTGAA | AGAGCGGCTGAAAATGCGTA |
| *OsWRKY23* | TCCAGTTCCTCTCCCAGTTCTAA | CACATTGTTCTCCTTTTCTTCCC |
| *OsWRKY72* | CACCACAAATCACATCTACTCCG | GCTGAAGGGAAGAGAGGTGAG |
| *OsNAC2* | AAAAACAACCGCATTGGCAG | AGTCCTCATCTCCTCTGTCTAATCC |
| *NYC1* | CATGCAACACCAACAAAAGG | GACCATTCCAGGAGAAGCAG |
| *NYC3* | TGTCGTTGCCATGTGAAGAT | TTGGTCACGCCACAAATCTA |
| *Osh36* | GCACGGAGGCGAACGA | TTGAGCGGTAGCACCCATT |
| *Osh69* | TTGAGCGGTAGCACCCATT | CCACAACACGGATAACTT |
| *Os157* | ACCCTAAAGTAAATGAAGTC | CCTGCTCTTGTCTTGTTA |
| *Osl85 F* | GAGCAACGGCGTGGAGA | GCGGCGGTAGAGGAGATG |
| *RCCR1* | CGCATTTCCTCATGGAATTT | CTTCTCACGCTGTTTGTCCA |
| *SGR* | AGGGGTGGTACAACAAGCTG | GCTCCTTGCGGAAGATGTAG |
| *Cab1R* | AGACGTTCGCCAAGAACC | GAGGAGCTCCGGGAAGAC |
| *Cab2R* | GTTCTCCATGTTCGGCTTCT | GACGAAGTTGGTGGCGTAG |
| *CHLD* | GGAAAGAGAGGGCATTAG | CAATACGATCAAGTAAGTGTT |
| *CHLH* | CTATACATTCGCCACACT | TATCACACAACTCCCAAG |
| *CHLI* | AGTAACCTTGGTGCTGTG | AATCCATCAACATTCAACTCTG |
| *OsHO1* | GACAGCAAGCTCGTCTTCG | GACCCTGTTCCTTGAACCATTC |
| *PORA* | ATCACCAAGGGCTACGTCTC | GAGTTGTTGTTCCAGCTCCA |
| *PSaA* | GAGATACCACTTCCTCAT | ACTAAGAAATTCTGCGTATT |
| *PSbA* | AAGTTTCTCTGATGGTATG | ATAGCACTGAATAGGGAA |
| *PR1a* | TTCATCACCTGCAACTACTCG | TGCATAAACACGTAGCATAGCAT |
| *PR1b* | GTGTGCGGGCACTACACG | CGGCTTATAGTTGCATGTGA |
| *PR5* | ATCGACGGCTACAACGTC | GTGTCTTGGTGTTGTCTTCG |
| *PR10* | CACCATCTACACCATGAAGC | AGCACATCCGACTTTAGGAC |
| *OsNCED1* | CTCACCATGAAGTCCATGAGGCTT | GTTCTCGTAGTCTTGGTCTTGGCT |
| *OsNCED2* | GGTATGGAAACGAGGATAGTGGTT | TGCTTATTGTTGTGCGAGAAGTTC |
| *OsNCED3* | CCCCTCCCAAACCATCCAAACCGA | TGTGAGCATATCCTGGCGTCGTGA |
| *OsNCED4* | TCCATCTCCTTCTCCCTCCTCCCA | CCTCGCACCCTGCTTGATCTTGCC |
| *OsNCED5* | ACATCCGAGCTCCTCGTCGTGAA | TTGGAAGGTGTTTTGGAATGAACCA |
| *LOX2* | GCATCCCCAACAGCACATC | AATAAAGATTTGGGAGTGACATATTGG |
| *AOS2* | CAATACGTGTACTGGTCGAATGG | AAGGTGTCGTACCGGAGGAA |
| *AOC* | AAGAGGAATCGAGGACAAGATATTTG | AAGCCTCTTCTTGTTCGGATCA |
| *OPR7* | GACCGCACTGACGAGTATGGT | CCACAGCCCTAGTTACCTCAAGTAG |

**Table S3** The chlorophyll a/b ratio of *bml* and WT at the tillering, heading stage and reproductive stage of rice.

| **Parameter** | **Chlorophyll a/b ratio** | |
| --- | --- | --- |
|  | ***bml*** | **WT** |
| Tillering Stage | 2.28± 1.10* | 1.22 ± 1.21 |
| Heading stage | 2.76± 0.107* | 2.01± 0.08 |
| Grain filling Stage | 1.97± 0.976* | 3.123± 1.9 |

Asterisk implies a significant difference at *p* < 0.05 using Tukey’s method.

**Table S4** The photosynthesis of *bml* and WT at the tillering stage and reproductive stage of rice.

| Parameter | Tillering Stage | | Reproductive Stage | |
| --- | --- | --- | --- | --- |
|  | *bml* | WT | *bml* | WT |
| Pn (µmol CO_2_ m^-2^s^-1^) | 13.593 ± 0.316^*^ | 18.728 ± 0.519 | 9.922 ± 0.452^*^ | 13.058± 0.876 |
| Gs(mol H_2_O m^-2^s^-1^) | 0.149 ± 0.007^*^ | 0.246 ± 0.005 | 0.102 ± 0.003^*^ | 0.184 ± 0.011 |
| Ci (µmol CO_2_ m^-2^s^-1^) | 213.324 ± 0.976^*^ | 220.751 ± 1.990 | 271.77 ± 1.168^*^ | 290.803 ± 0.556 |
| Tr (mmol CO_2_ m^-2^s^-1^) | 6.996 ± 0.904^*^ | 9.870 ± 0.949 | 2.960 ± 0.118^*^ | 3.820 ± 0.523 |

Pn -Photosynthetic rate; Gs - Stomatal conductance; Ci - Intercellular CO_2_ concentration and (d) Tr - Transpiration rate.
